# Supplementary material for: West Antarctic ice retreat and paleoceanography in the Amundsen Sea in the warm early Pliocene
Source: Nat Commun. 2025 Jul 1;16:5609. doi: 10.1038/s41467-025-60772-8 (PMC12219073; doi:10.1038/s41467-025-60772-8)
Supplement: Supplementary file 1 — Supplementary Information [file 41467_2025_60772_MOESM1_ESM.docx]

**West Antarctic ice retreat and deepwater formation in the Amundsen Sea in the warm early Pliocene**

Sandra Passchier, Claus-Dieter Hillenbrand, Sidney Hemming, Werner Ehrmann, Thomas Frederichs, Steve M. Bohaty, Ronald Leon, Olga Libman-Roshal, Lisbeth Mino-Moreira, Karsten Gohl, Julia Wellner

Supplementary Figures


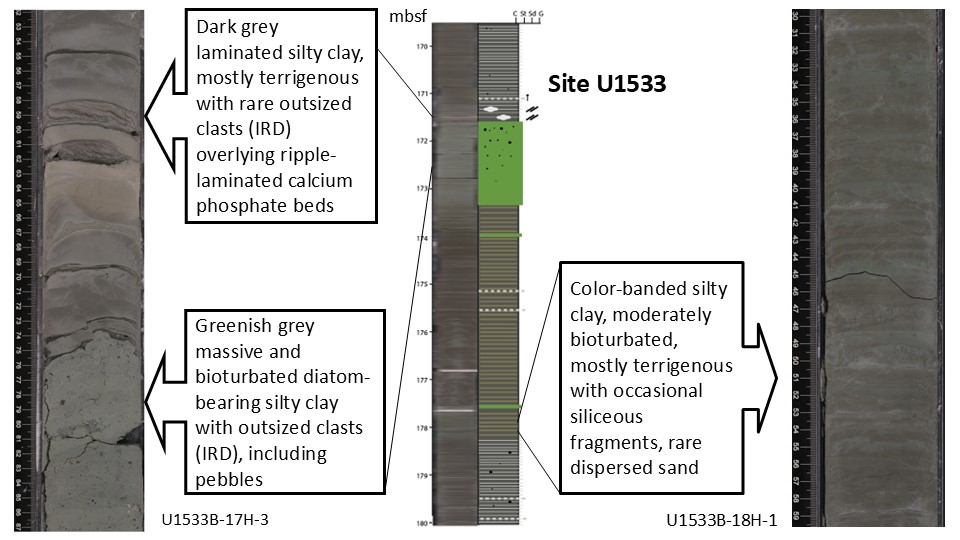


Supplementary Figure 1. Representative core section images with characteristic facies sequence for the lower Pliocene section of Sites U1532 and U1533, with from old to young, color-banded silty clay overlain by greenish grey silty clay, and dark grey laminated silty clay. Note the calcium phosphate bed and ripple cross-lamination in U1533B-17H-3 between 58 and 64 cm^1^.


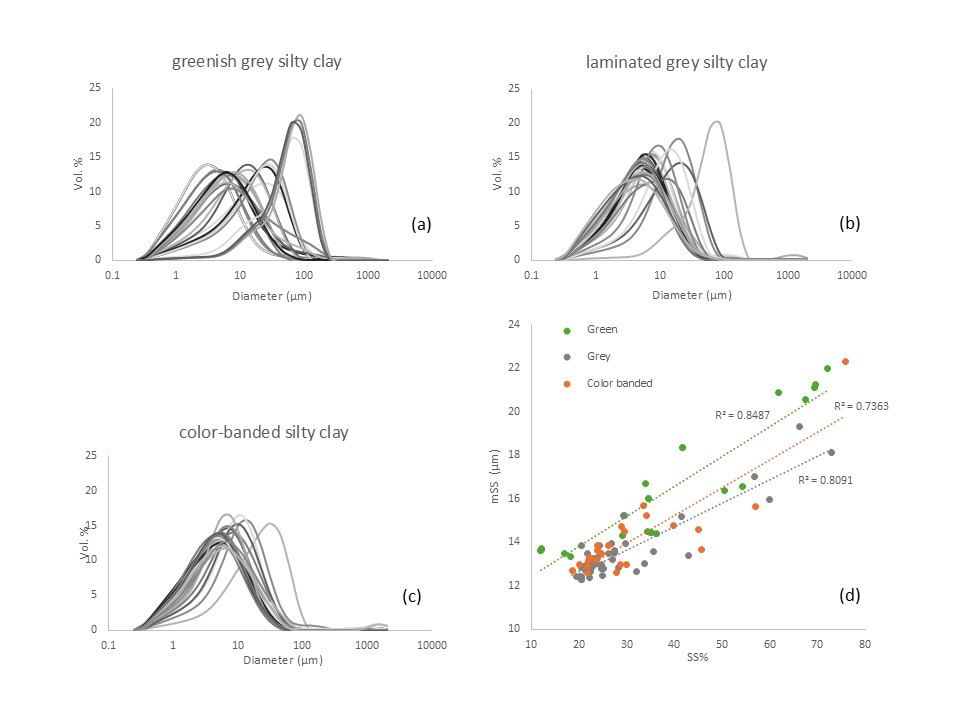
Supplementary Figure 2. Grain-size distributions for the three main facies, (a) greenish grey silty clay, (b) dark grey laminated silty clay, and (c) color-banded silty clay. Panel (d) displays a correlation plot of sortable silt percent (SS%) and mean sortable silt grain size (mSS). The strong correlation of greenish grey and laminated grey facies indicates that sediments were likely sorted by bottom currents, whereas the weak correlation for the color-banded facies suggests limited sorting by bottom currents.


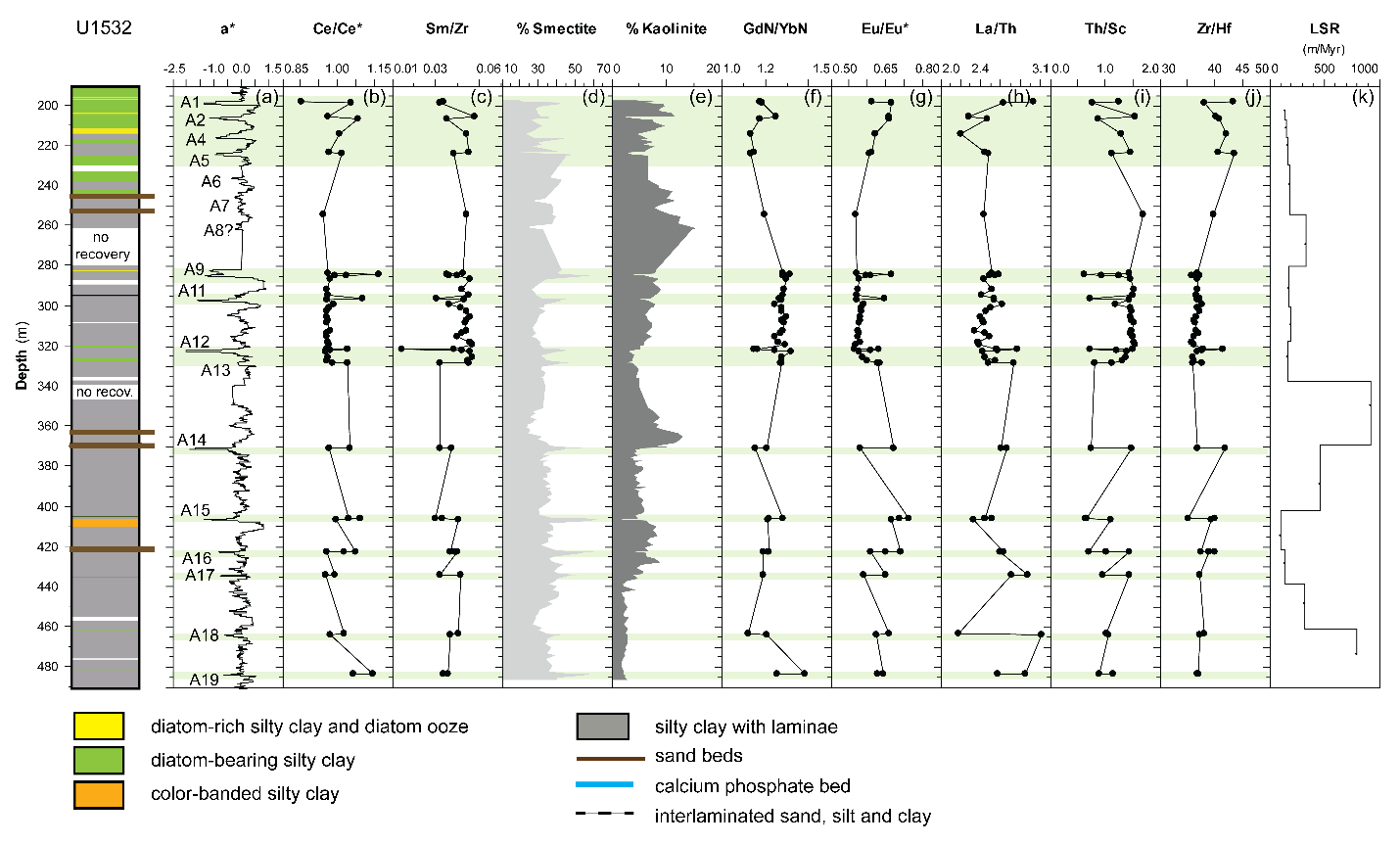


Supplementary Figure 3. Downcore distribution in Site U1532 of (a) a* red-green ratio^2^, (b) bulk sediment Cerium anomaly, (c) bulk sediment Sm/Zr ratio, (d) Smectite percent in clay mineral fraction, (e) Kaolinite, (f) bulk sediment GdN/YbN ratio, (g) bulk sediment Europium anomaly, (h) bulk sediment La/Th ratio, (i) bulk sediment Th/Sc ratio, (j) bulk sediment Zr/Hf ratio, and (k) linear sedimentation rate (LSR) in meter per Million year (m/Myr).


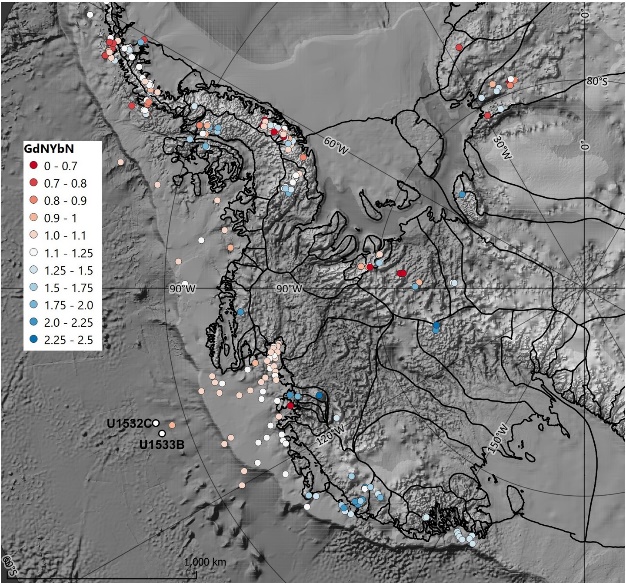


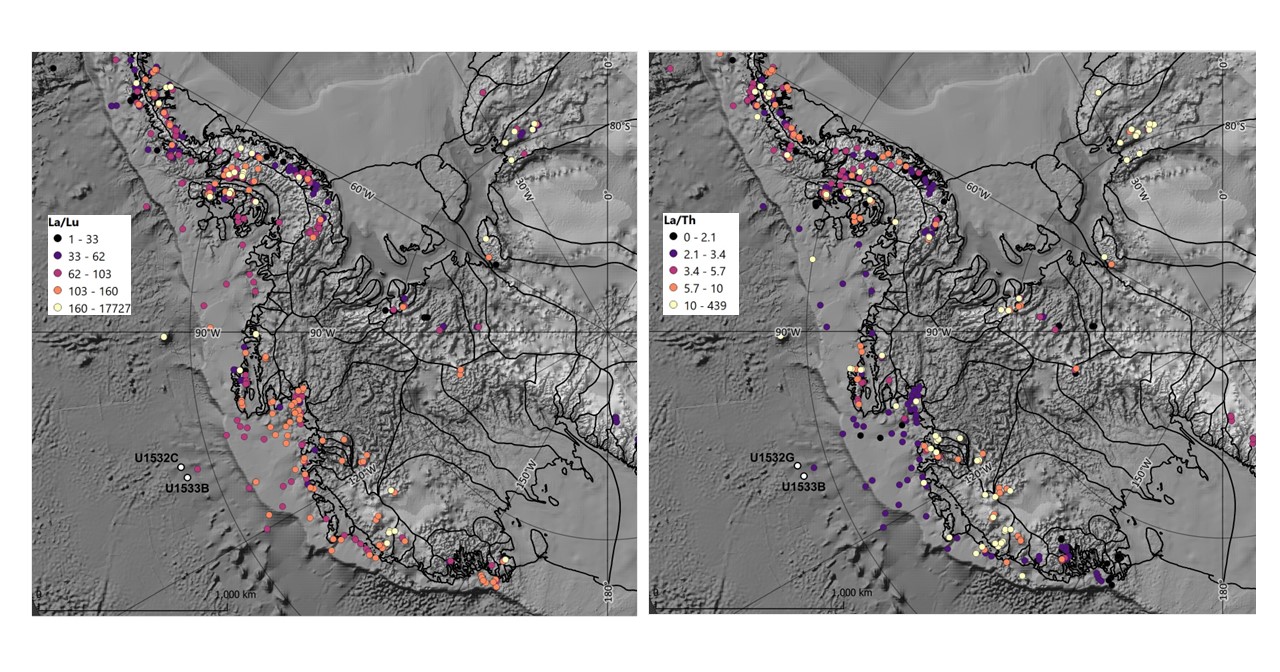

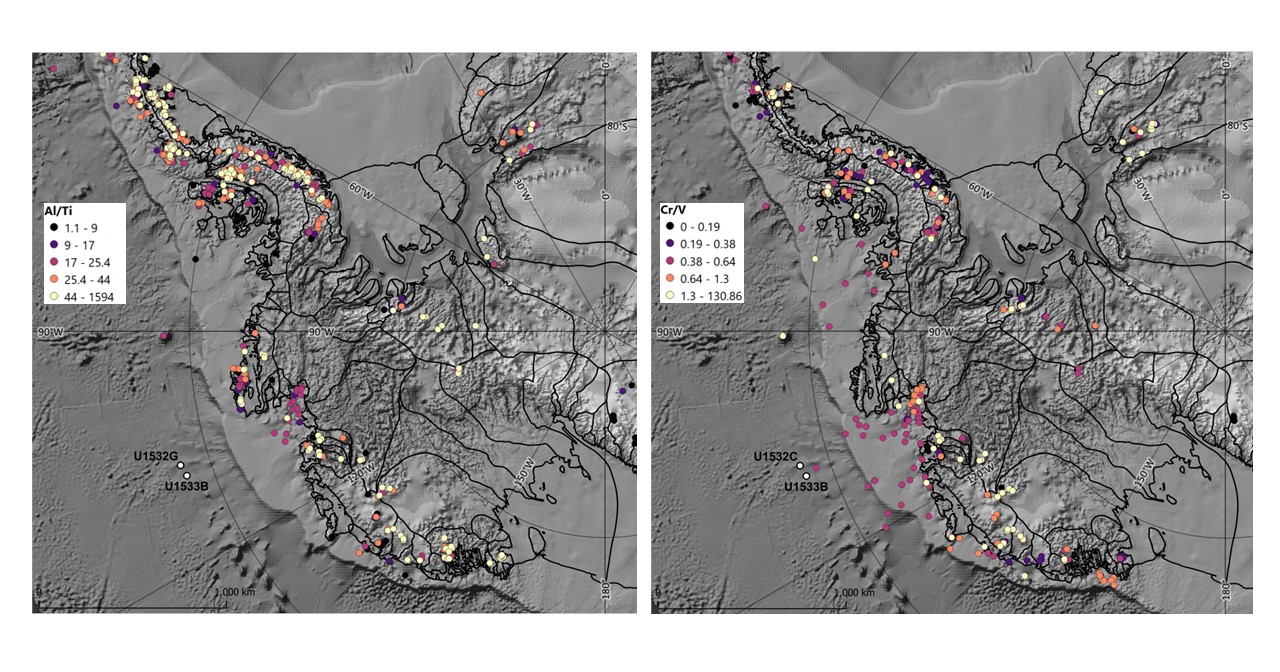

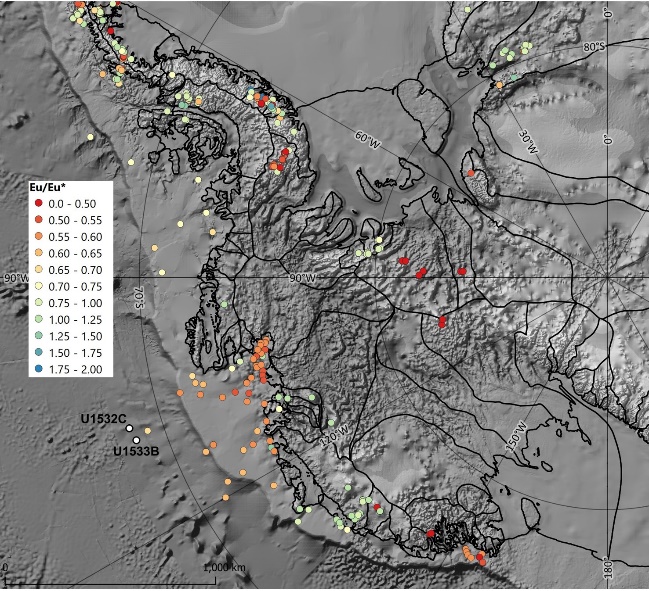


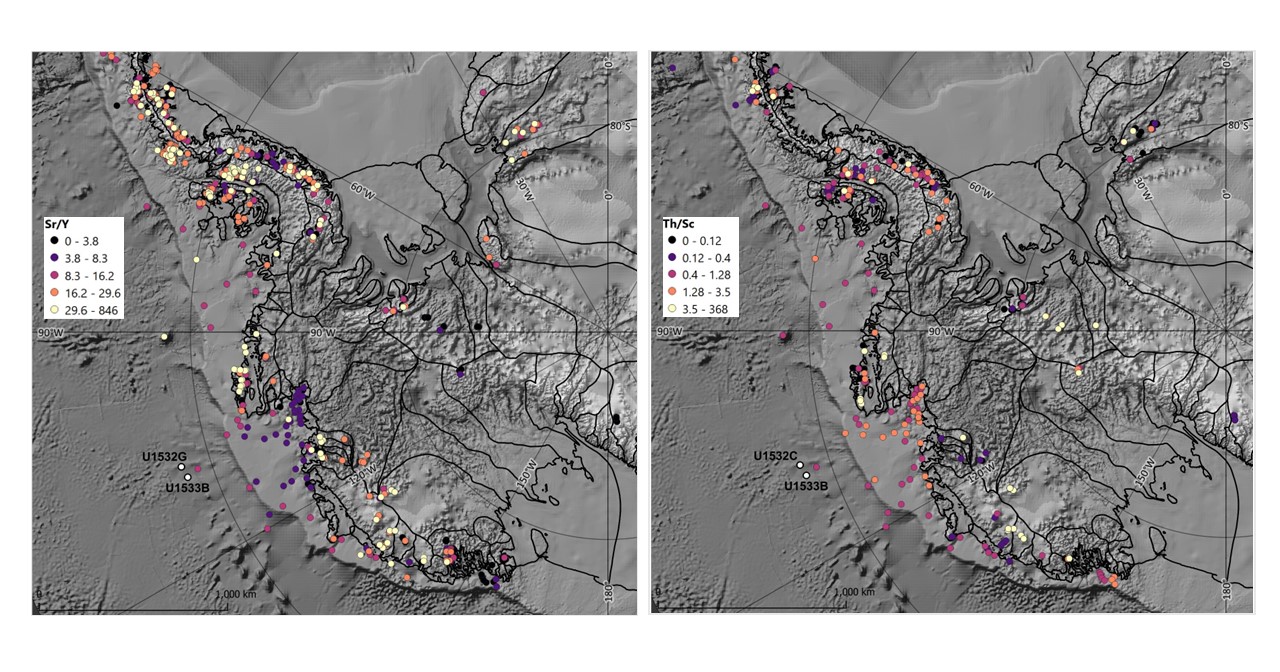


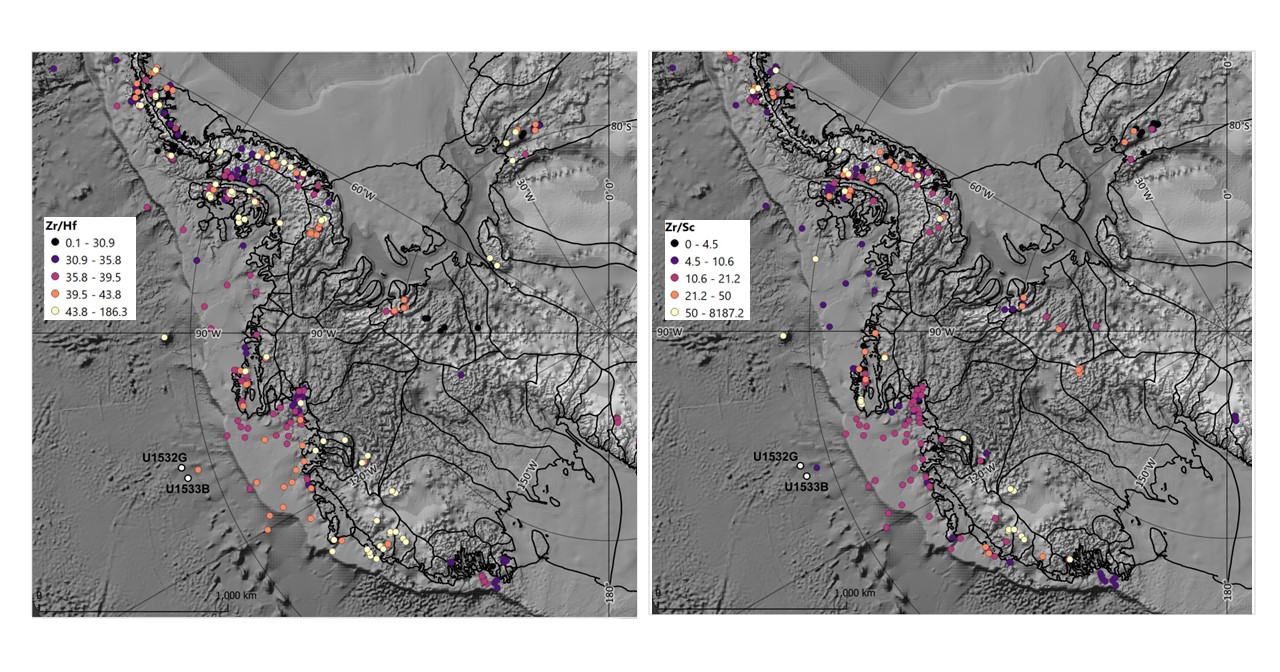

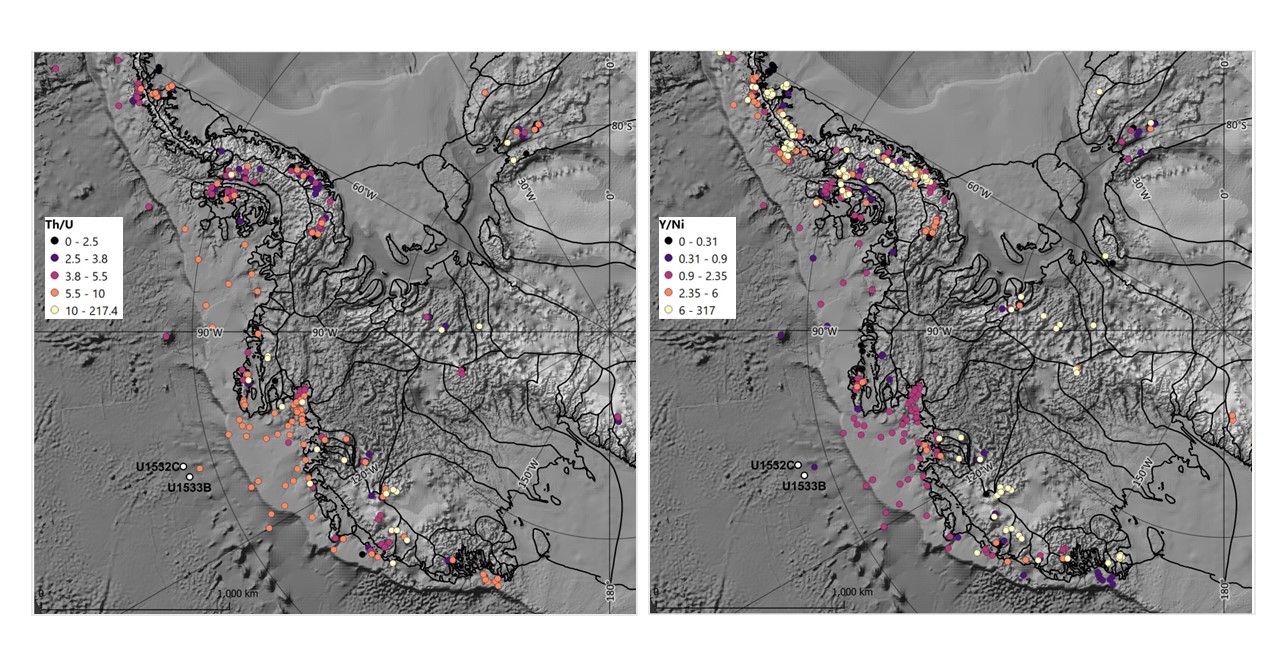

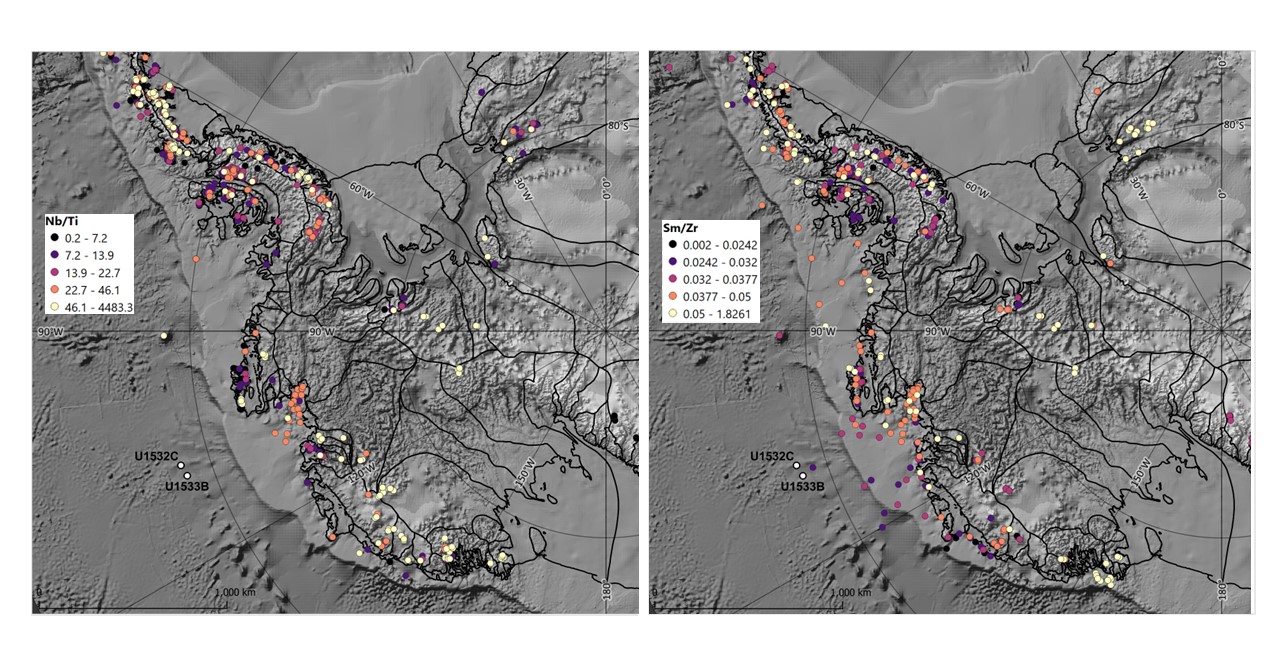
Supplementary Figure 4. Location of drill sites with elemental ratios derived from whole rock analyses. The Antarctic whole rock geochemical data was extracted from the database of Sanchez et al. (2021)^3^ and references therein^4-37^. The offshore geochemical data ^38-40^ was collected on the <63 µm grain-size fraction of seafloor sediments. The base map was produced using the geospatial data compilation Quantarctica^41^ in QGIS. The shaded relief map is based on BEDMAP2^42^.


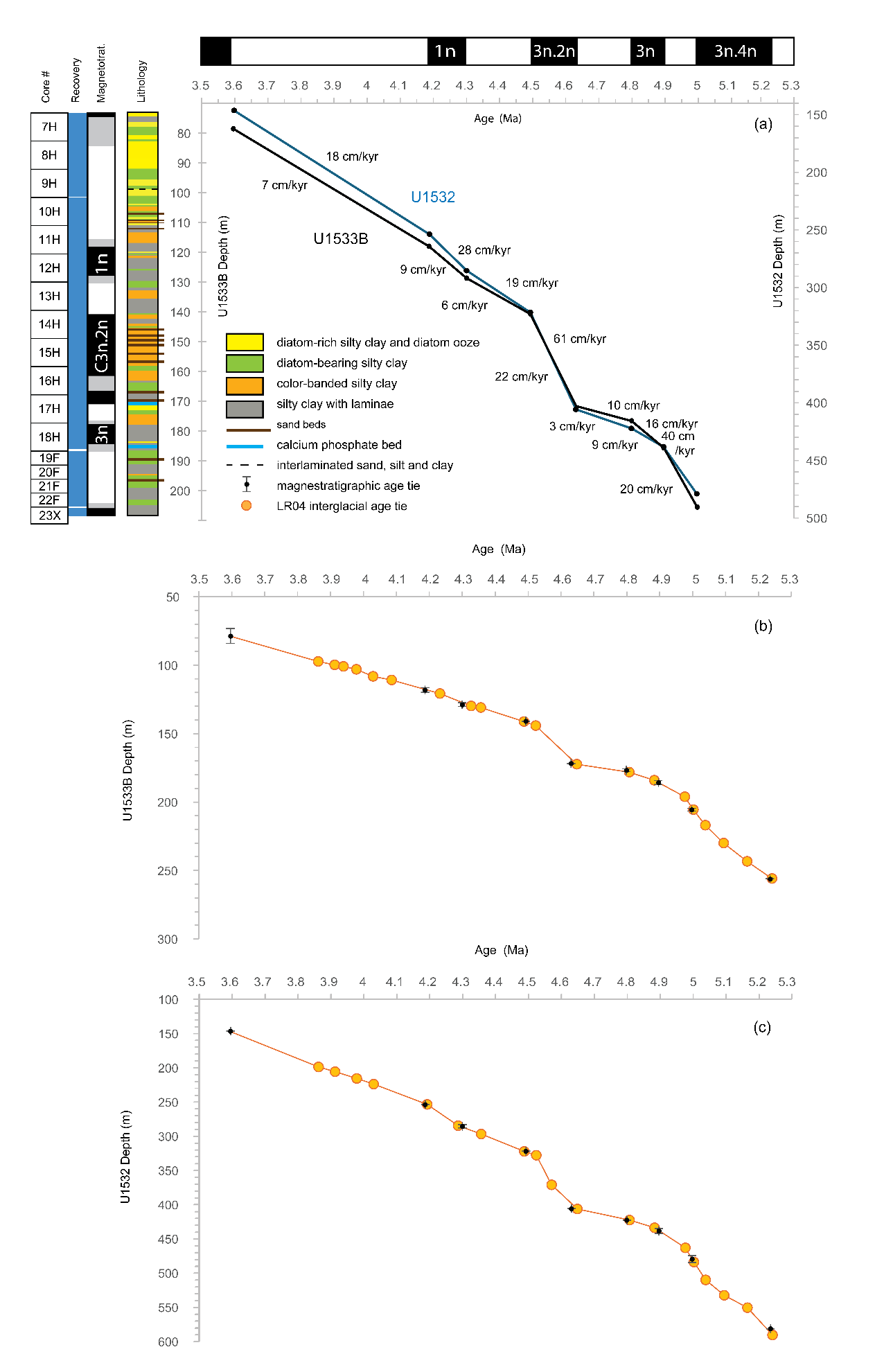


*(Supplementary Figure 5 caption on next page)*

Supplementary Figure 5. Linear age models based on the positions of paleomagnetic reversals and interglacial facies with (a) shipboard linear age models based on the positions of paleomagnetic reversals for IODP Sites U1532 and U1533^1-2^, (b) tuned age model for IODP Site U1533 based on the positions of paleomagnetic reversals^1-2^ and positions of tops of greenish grey units and/or a-star green-red color range minima tied to LR04 glacial-interglacial transitions, and (c) tuned age model for IODP Site U1532 based on the positions of paleomagnetic reversals^1-2^ and positions of tops of greenish grey units and/or a-star green-red color range minima tied to LR04 glacial-interglacial transitions. Depth in m CSF-A. Source data is provided for this Figure in Table 1.

1.

All samples





1. Biotite grains only





1. Hornblende grains only

Supplementary Figure 6. Density plots and histograms (10 Myr bins) for the ^40^Ar/^39^Ar ages of (a) All samples: hornblende, biotite and mica grains, (b) only biotite grains, and (c) only hornblende grains.

Supplementary References

1. Wellner, J., et al., Site U1533. Proceedings of the International Ocean Discovery Program, 2021. 379.
2. Wellner, J., et al., Site U1532. Proceedings of the International Ocean Discovery Program, 2021. 379.
3. Sanchez, G., et al., PetroChron Antarctica: A geological database for interdisciplinary use. Geochemistry, Geophysics, Geosystems, 2021. 22(12): p. e2021GC010154.107.
4. Care, B., The geology of Rothschild Island, north-west Alexander Island. British Antarctic Survey Bulletin, 1980. 50: p. 87-112.
5. Curtis, M.L., et al., Middle Cambrian rift-related volcanism in the Ellsworth Mountains, Antarctica: tectonic implications for the palaeo-Pacific margin of Gondwana. Tectonophysics, 1999. 304(4): p. 275-299.
6. Doubleday, P., et al., Allochthonous oceanic basalts within the Mesozoic accretionary complex of Alexander Island, Antarctica: remnants of proto-Pacific oceanic crust. Journal of the Geological Society, 1994. 151(1): p. 65-78.
7. Hart, S.R., J. Blijsztajn, and C. Craddock, Cenozoic volcanism in Antarctica: Jones mountains and Peter I island. Geochimica et Cosmochimica Acta, 1995. 59(16): p. 3379-3388.
8. Hart, S.R., et al., Hobbs Coast Cenozoic volcanism: implications for the West Antarctic rift system. Chemical Geology, 1997. 139(1-4): p. 223-248.
9. Hole, M., Post-subduction alkaline volcanism along the Antarctic Peninsula. Journal of the Geological Society, 1988. 145(6): p. 985-998.
10. Hole, M., P. Kempton, and I. Millar, Trace-element and isotopic characteristics of small-degree melts of the asthenosphere: Evidence from the alkalic basalts of the Antarctic Peninsula. Chemical Geology, 1993. 109(1-4): p. 51-68.
11. Hole, M. and W. LeMasurier, Tectonic controls on the geochemical composition of Cenozoic, mafic alkaline volcanic rocks from West Antarctica. Contributions to mineralogy and petrology, 1994. 117(2): p. 187-202.
12. Hole, M.J., Time controlled geochemistry of igneous rocks of the Antarctic Peninsula. 1986, Royal Holloway, University of London.
13. Leat, P.T., et al., Jurassic high heat production granites associated with the Weddell Sea rift system, Antarctica. Tectonophysics, 2018. 722: p. 249-264.
14. Leat, P.T., B.C. Storey, and R.J. Pankhurst, Geochemistry of Palaeozoic–Mesozoic Pacific rim orogenic magmatism, Thurston Island area, West Antarctica. Antarctic Science, 1993. 5(3): p. 281-296.
15. Lee, H.M., et al., The A-type Pirrit Hills Granite, West Antarctica: an example of magmatism associated with the Mesozoic break-up of the Gondwana supercontinent. Geosciences Journal, 2012. 16: p. 421-433.
16. LeMasurier, W., Shield volcanoes of Marie Byrd Land, West Antarctic rift: oceanic island similarities, continental signature, and tectonic controls. Bulletin of Volcanology, 2013. 75: p. 1-18.
17. LeMasurier, W.E., et al., Evolution of pantellerite-trachyte-phonolite volcanoes by fractional crystallization of basanite magma in a continental rift setting, Marie Byrd Land, Antarctica. Contributions to Mineralogy and Petrology, 2011. 162: p. 1175-1199.
18. LeMasurier, W.E., et al., Volcanoes of the Antarctic plate and Southern Ocean. Vol. 48. 1990: American Geophysical Union.
19. Macdonald, Leat, and Doubleday, On the origin of fore‐arc basins: New evidence of formation by rifting from the Jurassic of Alexander Island, Antarctica. Terra Nova, 1999. 11(4): p. 186-193.
20. McCarron, J. and J. Smellie, Tectonic implications of fore-arc magmatism and generation of high-magnesian andesites: Alexander Island, Antarctica. Journal of the Geological Society, 1998. 155(2): p. 269-280.
21. Moyes, A.B., The petrology and geochemistry of plutonic rocks across southern Graham Land, Antarctica. 1985, Royal Holloway, University of London.
22. Palais, J.M., et al., Magmatic and phreatomagmatic volcanic activity at Mt. Takahe, West Antarctica, based on tephra layers in the Byrd ice core and field observations at Mt. Takahe. Journal of Volcanology and Geothermal Research, 1988. 35(4): p. 295-317.
23. Panter, K.S., et al., Geochemistry of Late Cenozoic basalts from the Crary Mountains: characterization of mantle sources in Marie Byrd Land, Antarctica. Chemical Geology, 2000. 165(3-4): p. 215-241.
24. Panter, K.S., P.R. Kyle, and J.L. Smellie, Petrogenesis of a phonolite–trachyte succession at Mount Sidley, Marie Byrd Land, Antarctica. Journal of Petrology, 1997. 38(9): p. 1225-1253.
25. Riley, T.R., et al., Origins of large volume rhyolitic volcanism in the Antarctic Peninsula and Patagonia by crustal melting. Journal of petrology, 2001. 42(6): p. 1043-1065.
26. Rowley, P., Studies of the Geology and Mineral Resources of the Southern Antarctic Peninsula and Eastern Ellsworth Land, Antarctica, in US Geological Survey Professional Paper. 1988. p. 35.
27. Scarrow, J.H., et al., Antarctic Peninsula granitoid petrogenesis: a case study from Mount Charity, north-eastern Palmer Land. Antarctic Science, 1996. 8(2): p. 193-206.
28. Smellie, J.L., Geochemistry and tectonic setting of alkaline volcanic rocks in the Antarctic Peninsula: a review. Journal of Volcanology and Geothermal Research, 1987. 32(1-3): p. 269-285.
29. Storey, B., et al., Middle Jurassic within-plate granites in West Antarctica and their bearing on the break-up of Gondwanaland. Journal of the Geological Society, 1988. 145(6): p. 999-1007.
30. Strong, D., et al., Petlab: New Zealand’s national rock catalogue and geoanalytical database. New Zealand Journal of Geology and Geophysics, 2016. 59(3): p. 475-481.
31. Vennum, W.R., Igneous and metamorphic petrology of the southwestern Dana Mountains, Lassiter Coast, Antarctic Peninsula. Journal of Research of the US Geological Survey, 1978. 6(1): p. 95-106.
32. Vennum, W.R. and P.D. Rowley, Reconnaissance geochemistry of the Lassiter Coast intrusive suite, southern Antarctic Peninsula. Geological Society of America Bulletin, 1986. 97(12): p. 1521-1533.
33. Vennum, W.R. and B.C. Storey, Petrology, geochemistry, and tectonic setting of granitic rocks from the Ellsworth‐Whitmore Mountains crustal block and Thiel Mountains, West Antarctica. Gondwana Six: Structure, tectonics, and geophysics, 1987. 40: p. 139-150.
34. Wareham, C.D., I.L. Millar, and A.P. Vaughan, The generation of sodic granite magmas, western Palmer Land, Antarctic Peninsula. Contributions to Mineralogy and Petrology, 1997. 128: p. 81-96.
35. Weaver, S., J. Bradshaw, and C. Adams. Granitoids of the Ford Ranges, Marie Byrd Land, Antarctica. in International symposium on Antarctic earth sciences. 5. 1991.
36. Wever, H., B. Storey, and P. Leat, Peraluminous granites in NE Palmer Land, Antarctic Peninsula: early Mesozoic crustal melting in a magmatic arc. Journal of the Geological Society, 1995. 152(1): p. 85-96.
37. Zheng, G.-G., et al., Late Mesozoic–early Cenozoic intermediate–acid intrusive rocks from the Gerlache Strait area, Antarctic Peninsula: Zircon U–Pb geochronology, petrogenesis and tectonic implications. Lithos, 2018. 312: p. 204-222.
38. Carlson, A.E., et al., Absence of West Antarctic-sourced silt at ODP Site 1096 in the Bellingshausen Sea during the last interglaciation: Support for West Antarctic ice-sheet deglaciation. Quaternary Science Reviews, 2021. 261: p. 106939.
39. Simões Pereira, P., et al., The geochemical and mineralogical fingerprint of West Antarctica's weak underbelly: Pine Island and Thwaites glaciers. Chemical Geology, 2020. 550: p. 119649.
40. Simões Pereira, P., et al., Geochemical fingerprints of glacially eroded bedrock from West Antarctica: Detrital thermochronology, radiogenic isotope systematics and trace element geochemistry in Late Holocene glacial-marine sediments. Earth-Science Reviews, 2018. 182: p. 204-232.
41. Matsuoka, K., et al., Quantarctica, an integrated mapping environment for Antarctica, the Southern Ocean, and sub-Antarctic islands. Environmental Modelling & Software, 2021. 140: p. 105015.
42. Fretwell, P., et al., Bedmap2: improved ice bed, surface and thickness datasets for Antarctica. The cryosphere, 2013. 7(1): p. 375-393.
